# Fat Is Consistently Present within the Plantar Muscular Space of the Human Foot—An Anatomical Study

**DOI:** 10.3390/medicina58020154

**Published:** 2022-01-20

**Authors:** Joanna Tomlinson, Stefan Klima, Amélie Poilliot, Johann Zwirner, Niels Hammer

**Affiliations:** 1 Department of Anatomy, School of Biomedical Sciences, University of Otago, Dunedin 9016, New Zealand; ajpoilliot@outlook.com (A.P.); johann.zwirner@otago.ac.nz (J.Z.); 2 Department of Trauma, Orthopedic and Plastic Surgery, University Hospital of Leipzig, 04103 Leipzig, Germany; dr.stefan.klima@gmail.com; 3 HELIOS Park Hospital, Stümpellstraße 41, 04289 Leipzig, Germany; 4 Anatomical Institute, University of Basel, 4056 Basel, Switzerland; 5 Institute of Legal Medicine, University Medical Center Hamburg-Eppendorf, 22529 Hamburg, Germany; 6 Institute of Legal Medicine, University of Leipzig, 04103 Leipzig, Germany; 7 Institute of Macroscopic and Clinical Anatomy, Gottfried Schatz Research Center, Medical University of Graz, 8036 Graz, Austria; 8 Fraunhofer Institute for Machine Tools and Forming Technology (Fraunhofer IWU), Division of Medical Technology, 09126 Dresden, Germany

**Keywords:** aging, fat, foot, kinematics, sex, side

## Abstract

*Background and Objectives:* The foot comprises of active contractile and passive connective tissue components, which help maintain stability and facilitate movement during gait. The role of age- or pathology-related degeneration and the presence of fat within muscles in foot function and pain remains unclear. The existence of fat has to date not been quantified or compared between individuals according to age, sex, side or subregion. *Materials and Methods:* 18 cadaveric feet (mean age 79 years) were sectioned sagittally and photographed bilaterally. Fat in the plantar muscular space of the foot (PMSF) was quantified through the previously validated manual fat quantification method, which involved observing photographs of each section and identifying regions using OsiriX. Fat volume and percentage was calculated using a modified Cavalieri’s method. *Results:* All feet had fat located within the PMSF, averaging 25.8% (range, 16.5–39.4%) of the total PMSF volume. The presence of fat was further confirmed with plastination and confocal microscopy. *Conclusions:* These findings suggest that fat within the PMSF is a consistent but highly variable finding in elderly cohorts. Fat within the foot muscles may need to be considered a norm when comparing healthy and non-healthy subjects, and for therapeutic interventions to the foot. Further work is required to understand in detail the morphological and mechanical presence of fat in the foot, and compare these findings with pathological cohorts, such as sarcopenia. Additionally, future work should investigate if fat may compensate for the degeneration of the intrinsic muscles of the foot, with implications for both the use of orthotics and pain management.

## 1. Introduction

Physiological functioning of the foot–ankle complex is essential for the kinematics of the entire lower limb. Both active and passive components of the musculoskeletal apparatus, namely, muscles, bones and ligaments, help maintain foot stability and load distribution [1]. Similarly, the neurovascular system is involved in steering foot kinematics [1]. The active, passive and neural subsystems form the components of the foot core system (FCS) and contribute to maintaining foot function [1]. The foot’s intrinsic muscles are gaining increasing attention in injury prevention [2,3,4], as well as for musculoskeletal assessment [5,6] and rehabilitation [3,7,8] of lower limb disorders.

Current exercise protocols for the foot’s intrinsic muscles focus on muscular strength [2,9] and functional performance [10,11,12]. Generally, across the human body, when kinematic function is altered, it has been reported that decreased muscle mass and adipose tissue deposits are also found [13,14,15,16]. This likely also occurs in the foot. In cases where muscular atrophy is progressive and generalized across the body, this is known as sarcopenia. This may also contribute to frailty [14], as within the foot, decreased muscle strength is associated with an increased risk of falls [17].

Adipose tissue is omnipresent across the human body. It acts as an energy store [18], plays a role in structural support [19], shock-absorption [20], forms neurovascular pathways [19] and contributes to load distribution [21,22,23], homeostasis [18] and lipid metabolism [24]. Fat is also associated with pathologies such as diabetes [14] and pain [25], and, amongst other factors, the immune-endocrine function of adipose tissue is of central interest in the development of these pathological conditions. Fat also coincides with accelerating degenerative changes [26]. Structural muscular changes and fat within the foot have been noted using MRI [25,27,28,29,30,31,32,33,34] and ultrasound [5,35]. However, these methods have to date not yet quantified the fat within the foot across the population, nor have they proposed a specific role of adipose tissue. Understanding the topographical anatomy is firstly important before determining the role and changes in disease. An age-related increase in intramuscular fat has been noted in the lower limb [15] and back [36] muscles; nevertheless, it is unclear if such findings also exist for the foot. Little is known about the distribution of fat within the intrinsic muscular compartment of the foot, particularly the plantar aspect, and if there are inter-individual differences according to sex, side or region.

The purpose of this study was to assess the fat volume in the plantar muscular space of the foot (PMSF) in a population of elderly individuals. We stated the following hypothesis: Fat is present consistently in the PMSF of elderly individuals.

## 2. Materials and Methods

### 2.1. Specimens and Preparation

In total, 18 cadaveric feet from 15 cadavers (7 male, 11 female, 3 bilateral specimens, 79.3 ± 12.9 years mean age and standard deviation, and 49–94 years old age range) were included in this study. These tissues were donated by body donors under the 2008 Human Tissue Act of New Zealand to the University of Otago. The study was conducted in accordance with the Declaration of Helsinki and the protocol was granted ethical approval by the University of Otago Ethics Committee (ref: H17/20). All cadavers were embalmed using the Crosado embalming solution [37]. The feet were X-rayed laterally and then assessed for bony malformation by a clinician (SK) specialized in lower limb pathology, to determine if any specimens had a pathology atypical in an elderly cohort and should be excluded. Ten feet from nine cadavers included in the study had signs of osseous pathology common for the age of the body donors when passing, including osteoarthritis or ankle fracture. These specimens were excluded as they demonstrated small differences in percentages of fat (no known pathology vs. pathology, mean ± standard deviation, 25.8 ± 7.7 vs. 24.9 ± 5.9), which may be a result of pathology, but cannot be confirmed with this sample size or varied group. The remaining body donors had no lower limb pathology nor signs of pathological conditions in the X-rays or in dissection. No other specimens were excluded. These specimens were from body donors with varying body sizes (light, average and heavy) and very low to average muscle definition and size (measured using a 1–5 scale, with 1 being very low muscle definition and size and 5 being very good muscle definition and size). This resulted in 8 feet from 7 cadavers being included in our study (4 female, 3 male, 1 bilateral specimen, 84 ± 7.7 years mean age and standard deviation, and 73–94 years old age range).

This study followed the previously employed methodology employed by Poilliot et al. (2019) [38] and applied it to the feet. Specimens were firstly frozen at −80 °C and then sectioned in the sagittal plane using a 0.74-mm band saw (B16, Butcher Boy Machines, Selmer, TN, USA) into slices with an average thickness of 1.92 mm (±0.44 mm; range, 1.01 to 2.78 mm), as measured using digital calipers. These were then photographed against a 1 × 1-mm grid with a camera (Canon EOS 7D, Tokyo, Japan) attached to a fixed camera mount at a consistent distance of 500 mm between the specimen and objective. The image focus was set 2 mm above the scale paper, and manual magnification was chosen to suit the borders of all images of all series (400 × 400 mm). Both the medial and lateral aspects of each slice were photographed and included in the analysis. A total of 20–30 sections were included in the analysis from each specimen.

### 2.2. Quantification of Fat within the Plantar Muscle Compartments of the Foot

**Figure 1:**
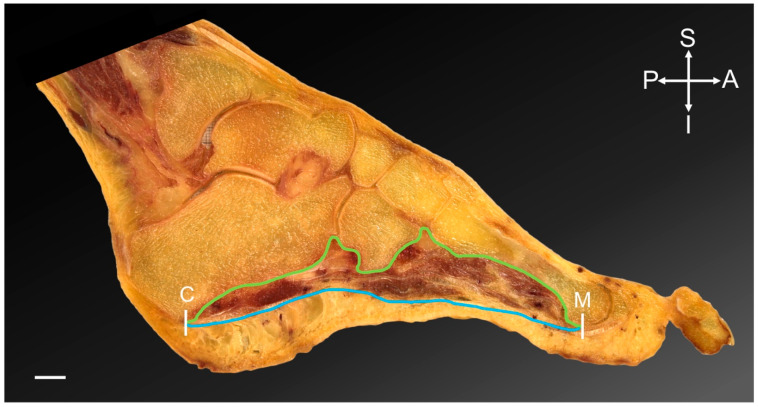
Foot section, sagittal plane. The region of interest of the plantar muscular space of the foot is highlighted. White lines indicate the anterior and posterior margins; the head of the metatarsal 1–5 (M) and the calcaneus (C). The blue line represents the inferior margin of the space with the plantar aponeurosis inferiorly. The green line indicates the superior boundary, which is the bony margins of the longitudinal arch of the foot. Scale bar = 10 mm. A = anterior; I = inferior; P = posterior; S = superior.

Images were firstly assessed for pin-cushion and barrel deformity using Adobe Illustrator CS6 (version 16.0.0, Adobe Systems Inc., San Jose, CA, USA) to detect image distortion. No deformity was noted. Images were then imported into OsiriX (version 11.0.2, Pixmeo SARL 2020, Geneva, Switzerland) to calculate the regions of interest (ROI) of each section, which were (1) the overall intrinsic area of the PMSF; and (2) the maximum area of adipose tissue within the initial PMSF area. The ROI boundaries and area were determined and drawn by one author (JT) trained in anatomy; these boundaries were determined according to the color and texture of the tissue within the image, with adipose tissue being yellow in appearance. This author circled around the borders of the PMSF and regions of fat within the PMSF to compute their area. The PMSF was defined by the bony margins of the foot and plantar aponeurosis (Figure 1). In sections where visibility of the metatarsals was compromised, an arched line was drawn, following the pattern of the previous section, to connect the base and head of the metatarsal. The ROI’s area was calibrated from pixels into cm^2^ using graph paper located behind the specimen. The fat ROIs were segmented in the same manner using color and texture to determine the location within the boundaries of the PMSF.

A modified version of Cavalieri’s Method, as described previously [38], was used to calculate volume of the PMSF and fat:

Vtotal=d(ΣAsurf)

This indicates that the sum of the area of each section (*Asurf*) was multiplied by the average distance (*d*) between sections. The average distance was calculated to be 1.33 mm, as both sides of each slice were observed in this study, according to the following formula:

$$d=\frac{slice\;thickness+bandsaw\;width}{2}$$

The percentage of fat within the PMSF was calculated using the following formula:

$$Total\;fat\%=\frac{Vfat}{VtotalPMSF}\times 100$$

Foot size is known to vary between individuals. This is a confounding variable on the dependent variable. The statistical results derived from comparing the percentage of fat between groups was used to form the basis of the conclusions rather than absolute values.

To compare the foot’s subregions, the available set of sections was divided into 3 groups. The most medial third of the mediolateral sections represented the medial compartment, the middle third represented the median and the lateral third of the sections represented the lateral aspect of the foot. In cases where the number of sections could not be divided into equal groups, the remaining section positioned between the lateral and middle regions and medial and middle regions was not counted. The total fat percentage of these regions was calculated using the formula describe above.

A subset of samples (n = 12) was reassessed by JT and assessed by another trained anatomist (AP)—to determine the intra- and inter-observer reliability of the method. The assessors were blinded to the demographics of the cadavers, to avoid bias and the other observer’s result. The first examiner was also blinded to their previous results through a 2-month time gap in assessing and reassessing the data.

### 2.3. Plastination and Confocal Microscopy

Fourteen foot sections from nine cadavers were randomly selected and plastinated using an epoxy resin mixture (E12/E1/AE20 in a ratio of 95:28:20 parts by weight; Biodur Products GmbH, Heidelberg, Germany). These were observed by JT to identify the fat and distribution of soft tissue. One of these sections was used to identify fat under a confocal laser scanning microscope (Zeiss LSM 710, Carl Zeiss Microscopy GmbH, Jena, Germany), with the thickness of the optical section set at 16.7 μm under 10× magnification. The plastination process results in collagen, elastin, myofilaments and neurofilaments being endogenously auto-fluorescent under 488 nm excitation [39].

### 2.4. Statistical Analysis

The data were processed and statistically analyzed using Microsoft Excel (version 16.16.25, Redmond, WA, USA), GraphPad Prism (version 7.0a, San Diego, CA, USA) and SPSS (version 27.0, IBM, Armonk, NY, USA).

Normal distribution was determined using a Shapiro–Wilk test. Correlations between fat volume and total PMSF volume were calculated, as well as between fat percentage and age. A perfect correlation was defined as equal to 1.0, very strong as ≥0.8, moderate as ≥0.6, fair as ≥0.3 and poor ≥0.1, based on Chan et al. (2003) [40]. *p* values of 0.05 or less were considered statistically significant. Values are displayed as the means ± standard deviations for normally distributed data and median and interquartile range (IQR) for data with a skewed distribution. Intra- and inter-observer reliability was assessed using Cronbach’s α and a two-way, mixed intra-class correlation coefficient (ICC), respectively, where a good result was set at ≥0.70, and ≥0.90 indicated an excellent correlation. Power analysis was performed using the UCSF sample size calculator [41], to determine the sample size required to indicate significance different results with significance and power levels set at 0.05 and 0.80, respectively.

## 3. Results

### 3.1. All Specimens Demonstrated Fat within the PMSF

**Figure 2:**
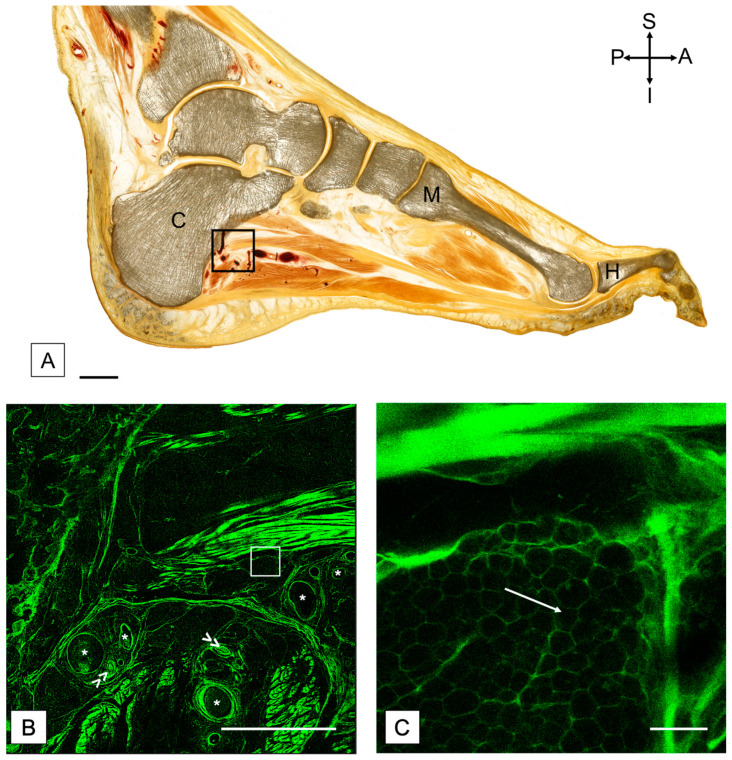
(**A**) Plastinated sagittal section of a foot with confocal images to demonstrate the adipose tissue. The black square indicates the region shown in (**B**). Scale bar = 15 mm. A = anterior; C = calcaneus; H = hallux; I = inferior; M = metatarsal; P = posterior; S = superior. (**B**) Confocal scan of a region distal to the calcaneus, including vessels. The white square represents the region shown in (**C**). (**C**) Confocal scan with higher magnification to highlight the adipose tissue. White arrow points towards adipose tissue present between slips of muscle in the foot. Scale in (**B**) = 5 mm, and scale in (**C**) = 200 μm. Asterisk (*) = blood vessel. Double arrow (>>) = nerve bundle.

Fat within the PMSF was consistently observed in all specimens of this study irrespective of body size and muscle tone (Table A1). Fat volume was variable and ranged between 7.1 and 40.1 mm^3^ (mean ± standard deviation, 21.3 ± 9.5 mm^3^) within a total PMSF volume of 43.4–101.8 mm^3^ (mean ± standard deviation, 80.3 ± 18.5 mm^3^). Fat accounted for 16.5–39.4% of the total volume of the PMSF (mean ± standard deviation, 25.8 ± 11.2%). Observation of plastinated sections and confocal microscopy further confirmed that fat is present within the foot (Figure 2). This adipose tissue appears to be interspersed between and within the ligaments and muscles of the foot.

Inter and intra-observer reliability analysis of the whole cohort (pathological and no known pathological) showed a Cronbach’s α of 0.93 for PMSF volume and 0.87 for fat volume, indicating excellent and good intraobserver reliability, respectively. An ICC of 0.93 for PMSF volume and 0.94 for fat volume were found, indicating excellent interobserver reliability.

### 3.2. Likely No Difference in Fat Percentage According to Foot Size

A positive correlation was noted between volume of fat and the volume of the PMSF (*r* = 0.75, *p* = 0.03), indicating there is likely no difference in fat percentage according to foot size.

### 3.3. Percentage of Fat Was Similar between Sexes, Sides and Subregions

The male mean and standard deviation fat volume percentage was 29.6 ± 11.1% and the female mean and standard deviation was 23.6 ± 5.0%. The mean and standard deviation fat volume percentage on left-sided specimens was 26.0 ± 6.7% and the mean and standard deviation for the right was 25.7 ± 9.6%. Furthermore, with the numbers available, a similar fat percentage was noted in the medial (mean ± standard deviation, 26.7 ± 9.8%), median (mean ± standard deviation, 23.7 ± 8.9%; median, 20.6%; IQR, 16.7–32.7%) and lateral (mean ± standard deviation, 26.1 ± 8.6%) regions of the foot.

## 4. Discussion

This study is the first to demonstrate that adipose tissue within the plantar muscular space of the foot seems to be a constant phenomenon in the elderly. Further to this, the given study is also the first to quantify the extent of adipose tissue within the foot across a cohort.

### 4.1. Adipose Tissue within the PMSF Is Likely a Consistent Observation in Elderly Individuals

In this study of an elderly cohort, adipose tissue was consistently present within the PMSF, as shown by image analysis using OsiriX, and was further confirmed with confocal microscopy. Previous literature also shows fat is detectable in clinical settings using MRI [25,27,29,30,31,32,33,34]. This study shows that the quantity of adipose tissue appears to be largely variable. This phenomenon may be important to consider when planning surgery, monitoring wound and bone healing, pain management and with the use of orthotics, yet further research is required to determine the role of this adipose tissue in the foot region clinically. The methodology employed in this study to quantify fat may be deemed as subjective; however, intra- and inter-rater reliability analysis indicated excellent agreement, therefore confirming that this method was robust and reproducible. In Crosado embalmed cadavers, which were used in this study, ligaments and tendons are typically white in appearance and muscle has a reddish hue [37], while adipose tissue is yellow; it is therefore unlikely that the fat was misidentified in the images. Crosado embalming is thought to produce ‘welled’ (i.e., enlarged) tissue [37]; therefore, these proportional baseline values may be useful for establishing pre- and post-mortem methods for detecting different biomechanical pathologies related to fat percentage. Further study is required to understand if other age groups exhibit fat within the PMSF, or what factors may contribute to its presence.

### 4.2. It Is Unclear Whether Fat within the PMSF Helps or Hinders Locomotion

Fat was present consistently in the PMSF of all individuals studied. Therefore, it potentially contributes to maintaining the FCS, as these specimens had no known foot pathology or signs of pathology within their X-rays. However, the specific role of peri-, intra- and intermuscular fat specifically within the foot is presently unclear. It may be important for adapting muscle function, or it could be related to dysfunction or inactivity [26,42]. Adipose tissue is observed in healthy muscle, such as the lower limb [15] and back [36]; however, as the muscle atrophies with dysfunction, inactivity and disrupted regeneration, the fat volume increases and accommodates the remaining space [43]. In particular, as shown in other regions of the body, the fat may provide support [19] to the structure and arches of the foot and withstand compressive forces [20] occurring at the foot during gait. Furthermore, fat may facilitate the gliding of the respective foot muscle layers relative to each other, while simultaneously providing space for neurovascular bundles [19]. Confocal microscopy confirmed the arrangement of adipose tissue in proximity to nerve and vascular bundles, suggesting that it plays a protective role. Fat within the foot likely plays many other roles, which may be determined with further biomechanical, immunohistochemical, kinematic and physiological studies.

Fat within the PMSF also may have a critical point at which pathological change begins or be unrelated to pathology. Adipose tissue is present in some lower limb muscles in elderly individuals even without being related to pathology—irrespective of BMI [15,44]. When comparing fat quantities in other anatomical regions found by other researchers to those in this given study, the ratio of fat with the quadriceps femoris muscle (age range, 70–79 years old) was lower than in the PMSF (age range, 49–94 years old) (10.0 ± 6.3% vs. 24.4 ± 6.7%) [15]. The baseline values of fat without a presenting pathology typical for the elderly population may be higher in the foot when compared to other regions. Changes in muscle composition alter the tensile stresses and elastic moduli of muscles [44] and also diminish muscular activation and contraction [42,45]. As a result, intra- and intermuscular fat is a known predictor of reduced balance, function, strength and mobility [42,44,46]. Therefore, these changes within the foot’s intrinsic muscles also likely increase the risk of biomechanical pathologies. Studies should compare the PMSF fat percentages between larger cohorts of individuals to determine if there is a critical point between asymptomatic fat and pathological fat. Considering possible alterations to the tissue characteristics (i.e., shrinkage or enlargement) due to fixation and the biomechanical effect(s) of plantar fat, these future studies should use modern imaging techniques to visualize fat in the weight-bearing foot of healthy volunteers.

### 4.3. Reciprocal Factors May Contribute to Fat in the PMSF

This study was unable to compare fat distribution according to age, sex and side due to the limited sample size. The results were underpowered and further studies are required to confirm this in the wider population. Power analysis of these preliminary findings indicates that a total of 54 feet would be required to compare sexes and 20,470 feet to compare sides; in turn, 754,278 feet would be required to compare ages between decades. This is likely not feasible to be performed with anatomic studies; therefore MRI, CT or ultrasound analysis would be required in future work.

Fat percentage was variable, and individual differences in percentage of fat may be a combined result of nutritional, physical and hormonal factors [14]. Since the age range of the individuals included in this study was limited (73–94 years old), the changes observed here may not be reflective of a wider age span. When referring to the previous literature, this indicates intermuscular fat may be a function of age, as age-related muscular changes are present in certain muscles [33] and regions of the foot [25], as well as other lower limb muscles [15]. Changes in muscular strength in relation to age have also been reported, as toe flexor strength decreases with age [47]. A larger cohort with a broader age range is required to test if there are discrete differences according to age.

Other factors may also contribute to presence of adipose tissue within the PMSF and should be studied further. Some of the donor’s patient records in the given analysis indicated conditions that may have resulted in altered mobility and lifestyle to the end that they were likely to have been sedentary for a period of time; i.e., lung cancer, heart failure or Alzheimer’s disease. Onset of these changes due to sedentary behavior appears to occur rapidly in other regions of the body, after as little as 3 days of inactivity or deconditioning: using the dry immersion model of severe muscle inactivity, individuals have demonstrated a reduction in muscle size by 11% and an upregulation of fatty infiltration markers [43]. Levels of exercise may also influence fat volumes [2,3,7,8,9,10,11,12] and therefore may have influenced the variable values reported. The role of pathology and lifestyle changes in relation to PMSF fat could not be studied due to limited information and the anatomical specimens’ varied causes of death in the given cohort.

Furthermore, other factors associated with muscle weakness and muscle fat deposits may be linked to the variability in fat noted within the PMSF, such as side dominance [48], increased levels of blood glucose [47], blood pressure [47] and sleeping time [47]. Body mass likely contributes to PMSF fat, as this has been noted in other body regions; for example, the thickness of the fat pad of the foot is reflective of a child’s body mass [49]. These presented variables could not be extensively studied due to the lack of donor information, highlighting the need for more clinical information from donors to inform future anatomical research on the topic. A sample size of 401 feet from participants or donors representing each body size, as well as muscle definition and size would be required to compare these variables. Based on the results available, the lack of age- or sex-specific differences suggest that the presence of fat within the PMSF involves highly complex interactions between numerous factors. Conversely, it is also probable that fat within the PMSF may be unrelated to demographic or environmental factors.

### 4.4. No Regional Differences in PMSF Fat Percentage Are Present in This Preliminary Cohort

Preliminary findings show that fat appears to be distributed similarly throughout the intrinsic muscular compartment of the plantar aspect of the foot within this cohort. However, a total of 6670 feet would be required to compare subregions and confirm this finding with sufficient power. Further study is required to determine if other spatial differences exist; i.e., differences in intra or intermuscular fat. Alternatively, differences could be present according to the depth within the muscle or in relation to the tendons, which are noted in the supraspinatus muscles [50,51]. This finding may indicate that fat deposits are related to structural–mechanical change, and such change may affect the plantar foot muscle composition homogeneously. Furthermore, if fat accumulation was metabolism-related, an equal distribution across the PMSF may be the result.

It remains unclear what biomechanical consequences may result from the increased fat percentage within the PMSF. Further study should investigate if a PMSF with a higher fat content may be at risk of having a weakened or fallen longitudinal arch, as a result of a differential force distribution, or any other structural foot or gait abnormalities. Ligament laxity, posterior tibial tendon degeneration or increased body weight are known to contribute to or act as a sign of pes planus (flat feet) [52,53], if uncompensated. It is unclear if fat within the PMSF would also contribute to this phenomenon. Further work should substantiate this in individuals with pes planus by performing biplanar X-ray to assess changes in arch support during gait, accompanied by correlations with fat content using imaging techniques.

### 4.5. Limitations

Limited anatomical specimens were available for the study and limited clinical information was available from the specimens included in this study; e.g., body mass index could not be obtained. The results may, therefore, be influenced by other confounding variables, such as nutritional, physical and hormonal factors, as well as the other factors mentioned earlier in this paper. It remains unclear if regional differences are present between the anterior and posterior and also superficial and deep aspects of the foot, as these were not compared in this study. This would have led to inaccurate divisions of the foot, as sections were cut in the sagittal plane. Additionally, the limited number of samples prevented statistical analysis of the differences in fat percentage according to foot size from being performed. Future work is required with a larger sample size to determine if differences exist. The use of averages in the calculations may have affected the accuracy of the figures reported to some extent, such as the average thickness of the slice. However, as the thickness of the section varied to a minute amount, this degree of error is thought to be negligible. Further to this, the calibration for each specimen was calculated according to the graph paper, which was located in a different plane to the region of interest circled. These inaccuracies are thought to potentially only minimally affect the results reported, as they were consistent.

## 5. Conclusions

Fat within the PMSF seems to be a consistent phenomenon within an elderly cohort. Further study with a larger sample size is required using modern imaging technologies, such as MRI, to substantiate these findings and determine the factors that contribute to fat within the PMSF, and if there is a critical point at which the presence of fat may be pathological.

## Data Availability

The corresponding author may be contacted to provide details.

## Appendix A

**Table A1:** Table demonstrating the fat percentage in individuals with different body sizes, and their muscle definition and size. Poor–average muscle definition and size = a score of 1 or 2; average–good muscle definition and size = a score of 3 or 4. NA = not applicable; SD = standard deviation.

	Light and Poor-Average Muscle Definition and Size	Average and Poor-Average Muscle Definition and Size	Average and Average-Good Muscle Definition and Size	Heavy and Poor-Average Muscle Definition and Size
**N**	3	3	1	1
**Mean**	26.72	28.53	26.60	17.60
**SD**	11.66	3.60	NA	NA
